# Gas Plasma-Treated Prostate Cancer Cells Augment Myeloid Cell Activity and Cytotoxicity

**DOI:** 10.3390/antiox9040323

**Published:** 2020-04-16

**Authors:** Sander Bekeschus, Verena Ressel, Eric Freund, Nadine Gelbrich, Alexander Mustea, Matthias B. Stope

**Affiliations:** 1 ZIK plasmatis, Leibniz Institute for Plasma Science and Technology (INP), 17489 Greifswald, Germany; verena-ressel@outlook.de (V.R.); Eric.freund@inp-greifswald.de (E.F.); 2 Department of Urology, University Medicine Greifswald, 17475 Greifswald, Germany; nadine.gelbrich@uni-greifswald.de; 3 Department of General, Visceral and Thoracic Surgery, University Medicine Greifswald, 17475 Greifswald, Germany; 4 Department of Gynecology and Gynecological Oncology, University Hospital Bonn, 53127 Bonn, Germany; alexander.mustea@ukbonn.de (A.M.); matthias.stope@ukbonn.de (M.B.S.)

**Keywords:** HL-60, immunomodulation, kINPen, LNCaP, PC3, plasma medicine, reactive oxygen species, ROS, THP-1

## Abstract

Despite recent improvements in cancer treatment, with many of them being related to foster antitumor immunity, tumor-related deaths continue to be high. Novel avenues are needed to complement existing therapeutic strategies in oncology. Medical gas plasma technology recently gained attention due to its antitumor activity. Gas plasmas act via the local deposition of a plethora of reactive oxygen species (ROS) that promote the oxidative cancer cell death. The immunological consequences of plasma-mediated tumor cell death are only poorly understood, however. To this end, we exposed two prostate cancer cell lines (LNCaP, PC3) to gas plasma in vitro, and investigated the immunomodulatory effects of the supernatants in as well as of direct co-culturing with two human myeloid cell lines (THP-1, HL-60). After identifying the cytotoxic action of the kINPen plasma jet, the supernatants of plasma-treated prostate cancer cells modulated myeloid cell-related mitochondrial ROS production and their metabolic activity, proliferation, surface marker expression, and cytokine release. Direct co-culture amplified differentiation-like surface marker expression in myeloid cells and promoted their antitumor-toxicity in the gas plasma over the untreated control conditions. The results suggest that gas plasma-derived ROS not only promote prostate cancer cell death but also augment myeloid cell activity and cytotoxicity.

## 1. Introduction

Despite recent improvements in cancer treatment, with many of them being related to foster antitumor immunity, tumor-related deaths continue to be high. With over 1.5 million new cases worldwide in 2018 and a high mortality rate [1], this is also seen in prostate cancer patients. While the 5-year survival rate of loco-regional prostate cancer is nearly 100%, it drops to 31% in the case of tumor metastasis [2]. In the US, prostate-cancer related death ranks second in men behind lung cancer. Standard treatment for loco-regional prostate cancer is surgery or radiation, while metastatic prostate cancer is targeted using androgen deprivation therapy as first-line treatment, with docetaxel as an emerging drug [3]. However, the disease is still fatal in many patients, exemplifying the need for novel therapeutic avenues against prostate cancer.

Medical gas plasma technology recently gained attention due to its antitumor activity against many types of cancers [4,5,6]. Specifically, plasma was shown to inactivate, for instance, malignant melanoma [7], squamous cell carcinoma [5,8], lung cancer [9], colon cancer [10], pancreatic cancer [11], osteosarcoma [12], glioblastoma [13], and hepatocellular carcinoma [14]. The technology uses electrical discharge to partially ionize a neutral gas, and is designed in a way that it promotes biomedical effects in cells and tissues without thermal harm [15]. Instead, the gas plasmas act via the local deposition of a plethora of reactive oxygen species (ROS) [16] that promote cancer cell death [17]. Previous studies suggested that plasma-induced tumor toxicity also has an immunogenic component [18,19,20], leading to antitumor immunity in vivo [21,22,23]. Specifically, it was observed that plasma treatment leads to an increased expression of calreticulin, a protein that dictates the immunogenicity of cells [24]. In vivo, plasma treatment not only limits tumor growth but also is associated with an increase in tumor-infiltrating leukocytes [25,26]. Prostate cancer also has an immunological dimension [27], and especially myeloid cells are frequently high-jacked by tumor cells to render them into tumor-promoting phenotypes [28].

To this end, we here investigated not only the cytotoxic potential of medical gas plasma technology in targeting human prostate cancer cells but also the immunomodulatory consequences of the latter towards human myeloid cells. Using both supernatant and co-culture assay systems, we identified an augmentation of myeloid cell activity and cytotoxicity towards gas plasma-treated prostate cancer cells in vitro.

## 2. Materials and Methods

### 2.1. Cell Culture

The prostate cancer cell lines LNCaP (ATCC, Manassas, VA, USA; ATCC CRL-1740) and PC3 (ATCC CRL-1435) as well as the myeloid cell lines THP-1 (ATCC TIB-202) and HL-60 (ATCC CCL-240) were used in this study. The prostate cancer cell lines were retrieved from metastatic lesions, with LNCaP as an androgen-sensitive cell line derived from a lymph node metastasis and the androgen-insensitive PC3 cell line derived from bone metastasis. The cells were cultured in Roswell Park Memorial Institute (RPMI) 1640 medium supplemented with 10% fetal bovine serum, 2% glutamine, and 1% penicillin and streptomycin (all Sigma-Aldrich, Taufkirchen, Germany). Cells were sub-cultured twice or thrice per week and maintained under standard culture conditions (37 °C, 5% CO_2_, and 95% humidity).

### 2.2. Gas Plasma Treatment of Tumor Cells

The atmospheric pressure plasma jet kINPen (neoplas tools, Greifswald, Germany) was operated at 3.0 standard liters of argon per minute and 1.1 MHz. Quality control of the plasma generation was done as previously described [29]. A thorough description of the physical principles of plasma jets was given recently [30]. For cell growth kinetics, 3 × 10^4^ (LNCaP) or 1 × 10^4^ (PC3) cells in 500 µL of fully supplemented cell culture medium were seeded into 24-well plates and exposed to different plasma treatment times. For the generation of tumor cell culture supernatants, 2 × 10^5^ cells in 500 µL of fully supplemented cell culture medium were seeded into 24-well plates and exposed to plasma. In initial experiments, argon gas only (plasma: off) treatment was used as a mock control. Argon is an inert gas with no known direct biological effects.

### 2.3. Tumor Cell Supernatant Incubation of Myeloid Cells and Setup of Co-Culture Assays

Four hours after plasma treatment, tumor cell culture supernatants were collected, centrifuged at 1000× *g* for 5 min to discard residual cells and debris, and stored at −20 °C until use. For experiments, 80 µL of this supernatant was added to wells of a flat-bottom 96-well plate (Eppendorf, Hamburg, Germany). To each well, 20 µL of a cell suspension containing 1 × 10^4^ of either THP-1 or HL-60 cells was added. The myeloid cells were incubated for up to 96 h. The Eppendorf 96-well plates have an outer rim that was filled with deionized water to prevent excessive evaporation from the outer wells, as observed with extensive culture durations. For the co-culture of prostate cancer and myeloid cells, the 96-well plates were coated with 0.01% poly-l-lysine. 2.5 × 10^4^ (LNCaP) or 1.25 × 10^4^ (PC3) in 100 µL of cell culture medium cells were labeled with trace violet (Thermo Fisher Scientific, Dreieich, Germany) before being added to each well. The cells were exposed to the gas plasma and incubated for another 30 min in the incubator. Subsequently, 2.5 × 10^4^ or 1.25 × 10^4^ THP-1 or HL-60 cells were labeled with cell trace red (Thermo Fisher Scientific, Dreieich, Germany) and added to the tumor cells together with sytox green (Thermo Fisher Scientific, Dreieich, Germany) for the identification of terminally dead cells.

### 2.4. Cell Counting, Metabolic Activity, and Cell Viability

For the counting of cells in growth kinetic experiments, a CASY cell counter and analyzer model TT (Roche Applied Science, Mannheim, Germany) with a 150 μm capillary was used. For analyzing the metabolic activity of cells, resazurin (100 µM; Alfa Aesar, Haverhill, MA, USA) was added. The non-fluorescent resazurin is converted intracellularly only in metabolically active cells via an NADPH-dependent reduction to the fluorescent product resorufin. After 2 h of incubation, resorufin fluorescence was quantified using a microplate reader (F200; Tecan, Männedorf, Switzerland) at λ_ex_535 nm and λ_em_590 nm. To determine the absolute number of viable myeloid cells, flow cytometry (CytoFLEX S; Beckman-Coulter, Brea, CA, USA) was used. Live-dead discrimination was done by the addition of 4′,6-diamidino-2-phenylindole (DAPI, 1 µM; Sigma-Aldrich, Taufkirchen, Germany), and only live cells were gated for subsequent analysis.

### 2.5. Analysis of Reactive Oxygen Species (ROS), Oxidation, and Mitochondria

Hydrogen peroxide (H_2_O_2_) was quantified using the amplex ultra red (Thermo Fisher Scientific, Dreieich, Germany) assay according to the manufacturer’s instructions as described in detail before [29]. To analyze the generation or deposition of ROS to the intracellular compartment, the myeloid cells were stained with either chloromethyl 2′,7′-dichlorodihydrofluorescein diacetate (CM-H_2_DCF-DA, 1 µM; Thermo Fisher Scientific, Dreieich, Germany) converted intracellularly to the fluorescent dichlorodihydrofluorescein (DCF) or 3′-(p-aminophenyl) fluorescein (APF, 1 µM; (Thermo Fisher Scientific, Dreieich, Germany) before the addition of tumor cell culture supernatants to 2 × 10^4^ labeled myeloid cells per well. Subsequently, the mean fluorescent intensity (MFI) of the dyes in the cells was analyzed by flow cytometry. To analyze the effect of the supernatants on the mitochondrial membrane potential, the myeloid cells were labeled with mitotracker orange (MTO, 1 µM; Thermo Fisher Scientific, Dreieich, Germany) before the addition of the tumor cell culture supernatants, and the MFI of MTO was analyzed using flow cytometry. In all assays, DAPI was used to gate on the live-cell population only for analysis of oxidation and mitochondria.

### 2.6. Cell Surface Marker Analysis

To analyze the expression of several myeloid cell surface markers simultaneously, multicolor flow cytometry was used. At 96 h, the myeloid cells were harvested into 96-well v-bottom plates using accutase. The cells were washed and stained against a selection of the monoclonal antibodies used in this study for targeting several cell surface receptors (Table 1). DAPI was added to quantify the MFI of each marker only in the live-cell population. After 15 min of incubation at room temperature in the dark, the cells were washed and acquired using a CytoFLEX S cytometer (Beckman-Coulter, Brea, CA, USA). The same procedure was performed for both the monoculture and co-culture assay systems. Discrimination of myeloid cells from tumor cells was done based on CD55 vs. HLA-ABC expression (THP-1 cells had higher baseline intensities for both parameters compared to prostate cancer cells) for THP-1 cells, and based on forward-scatter (tumor cells had higher signal intensities) vs. CD32 (HL-60 cells had higher signal intensities) for HL-60 cells.

### 2.7. Quantification of Chemokines and Cytokines

The quantification of chemokines and cytokines was performed in supernatants derived from prostate cancer cells (4 h after plasma treatment), myeloid cells incubated with and without supernatants of prostate cancer cells (at 96 h), and co-cultures of myeloid cells with cancer cells (at 96 h). To this end, a 12-target, bead-based sandwich immunoassay (LegendPLEX; BioLegend, London, UK) was utilized. Quantification was done against an internal standard for chemokine (C-C motif) ligand 17 (CCL17), chemokine (C-X-C motif) ligand 1 (CXCL1), CXCL10, interferon-gamma (IFNγ), interleukin 1-beta (IL1β), IL6, IL8, IL10, IL12p70, tumor growth factor-beta (TGFβ), tumor necrosis factor-alpha (TNFα), and vascular endothelial growth factor (VEGF).

### 2.8. Live-Cell High Content Imaging

Prostate cancer cells were labeled with cell trace violet (1 µM; Thermo Fisher Scientific, Dreieich, Germany) and cultured together with cell trace red-labeled myeloid cells (1 µM; Thermo Fisher Scientific, Dreieich, Germany) as described above for co-culture. Sytox green (0.1 µM; Thermo Fisher Scientific, Dreieich, Germany) was added for the detection of terminally dead cells. Live-cell imaging was performed using an Operetta CLS (PerkinElmer, Hamburg, Germany) high content imaging device equipped with a CO_2_ and temperature control chamber. Protection from evaporation was ensured since the rim of the 96-well plate was filled with deionized water. Imaging was performed at 24 h, 48 h, 72 h, and 96 h post plasma exposure using a 20× (NA 0.4) air objective (Zeiss, Jena, Germany) for 25 fields of view per well, time point, and channel. Channels were brightfield, digital phase contrast, cell trace violet (λ_ex_405 nm, λ_em_465 nm), sytox green (λ_ex_475 nm, λ_em_525 nm), and cell trace red (λ_ex_630 nm, λ_em_680 nm). A total of 25,000 images were acquired and analyzed in this study.

### 2.9. Statistical and Software Analysis

Statistical analysis was performed using prism 8.4 (Graphpad Software, San Diego, CA, USA). Either *t*-test, ratio-paired *t*-test, or one-way analysis of variances with *Dunnett* posthoc testing was used. The level of significance is indicated as follows: *p* < 0.05 (*), *p* < 0.01 (**), and *p* < 0.001 (***). At least three independent experiments with several technical replicates each were done for each assay. Flow cytometric analysis and fluorescence compensation were done using Kaluza analysis 2.1.1 (Beckman-Coulter, Brea, CA, USA). About 2000 individual measurements were analyzed in the present study. Quantitative image analysis was performed using Harmony 4.9 (PerkinElmer, Hamburg, Germany). The cells were segmented based on their digital phase-contrast signal, and the type of cell was determined by using a threshold on relative signal intensities from cell trace violet or cell trace red for prostate cancer and myeloid cells, respectively.

## 3. Results

### 3.1. The Plasma Jet Treatment Decelerated Prostate Cancer Cell Growth

**Figure 1:**
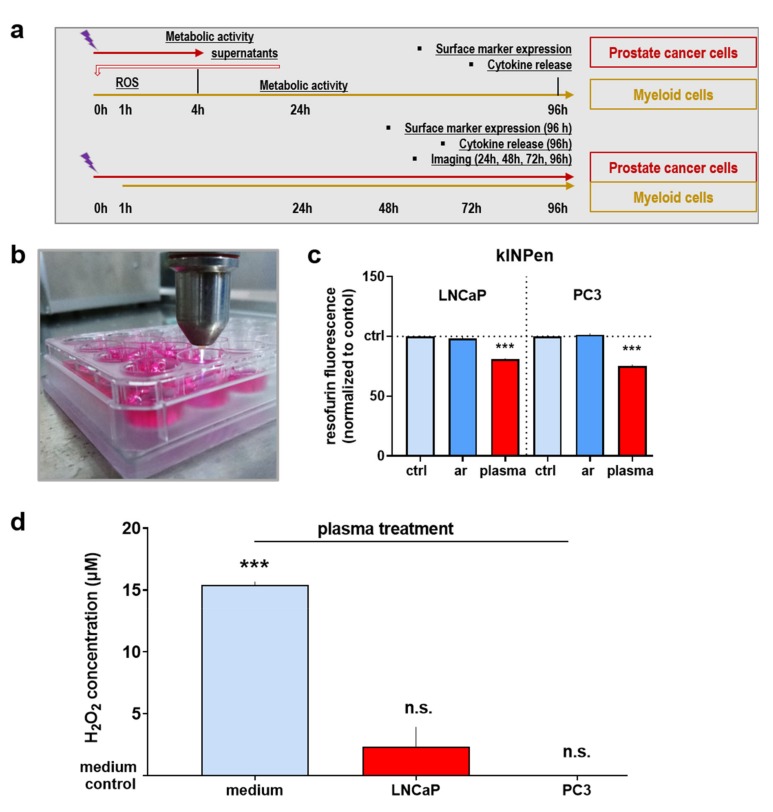
The kINPen gas plasma treatment decelerated the growth of prostate cancer cells. (**a**) outline of the experimental procedures and time lines performed in this study; (**b**) image of the kINPen atmospheric pressure argon plasma jet treating cells in a well of a 24-well plate; (**c**) metabolic activity of LNCaP and PC3 cells 4 h after kINPen plasma treatment; (**d**) residual reactive oxygen species (ROS) (H_2_O_2_) 1 h after kINPen plasma treatment of LNCaP and PC3 cells as well as fully supplemented cell culture medium compared to untreated medium control. Data are presented as mean ± SEM of three independent experiments. Statistical analysis was performed using one-way analysis of variances with posthoc testing after *Dunnett*. The level of significance is indicated as follows: *p* < 0.001 (***). ctrl = control; ar = inert argon gas mock treatment (plasma off).

Medical gas plasma treatment using the kINPen atmospheric pressure plasma jet was performed against prostate cancer cells in vitro. Two experimental schemes were used to identify the potential effects of that treatment in prostate cancer cells on myeloid cells (Figure 1a). In the first setup, prostate cancer cells were exposed to the plasma, and the cell culture supernatants were collected after four hours and added to myeloid cells. At 1 h, immediate effects such as ROS production and mitochondrial activity were determined. At 24 h, the metabolic activity and cell proliferation of the myeloid cells were investigated. At 96 h, the cell surface marker expression profile and cytokine release were assessed. In the direct co-culture approach, the prostate cancer cells were exposed to the plasma, and myeloid cells were added 1 h later. The co-cultures were imaged at 24 h, 48 h, 72 h, and 96 h, with an additional investigation of the cell surface marker profile of myeloid cells and the cytokine release in the supernatants of the co-cultures at 96 h. An image of the plasma treatment procedure is shown in Figure 1b. Based on previous studies, it was hypothesized that the plasma treatment decelerates tumor cell growth. Investigating the impact on the metabolic activity of the cancer cells 4 h after plasma treatment, the treatment led to a significant reduction in both prostate cancer cell lines investigated (Figure 1c). The plasma labeling denotes cells that were treated with the gas plasma directly. The argon control indicates conditions where the inert argon gas was not ignited into plasma but instead was merely blown onto the cell suspension as a mock treatment to exclude any biological effects of the noble gas alone. Similar to other plasma jets, the kINPen generates ROS in the plasma gas phase that subsequently diffuse into the treated liquid [31]. From there, the species further diffuse to cells [16] with some of the species possibly accumulating in the cytosol, as has been suggested for H_2_O_2_ passing through aquaporin channels in the membrane [32]. As the purpose was to investigate the immunomodulatory potential of supernatants from the untreated and plasma-treated prostate cancer cells, we also investigate residual ROS in these suspensions. Only a minor presence of ROS was observed in the plasma-treated LNCaP cultures 1 h after exposure, while residual ROS were absent for plasma-treated PC3 cells at 1 h (Figure 1d). At 4 h after plasma treatment, supernatants of the prostate cancer cells were collected and used for subsequent experiments with myeloid cells. Altogether, plasma treatment decelerated prostate cancer cell growth, and the majority of plasma-derived ROS have reacted with the cancer cells.

### 3.2. Prostate Cancer Cell Supernatants Affected the Intracellular Oxidative Milieu and Metabolic Activity of Myeloid Cells

**Figure 2:**
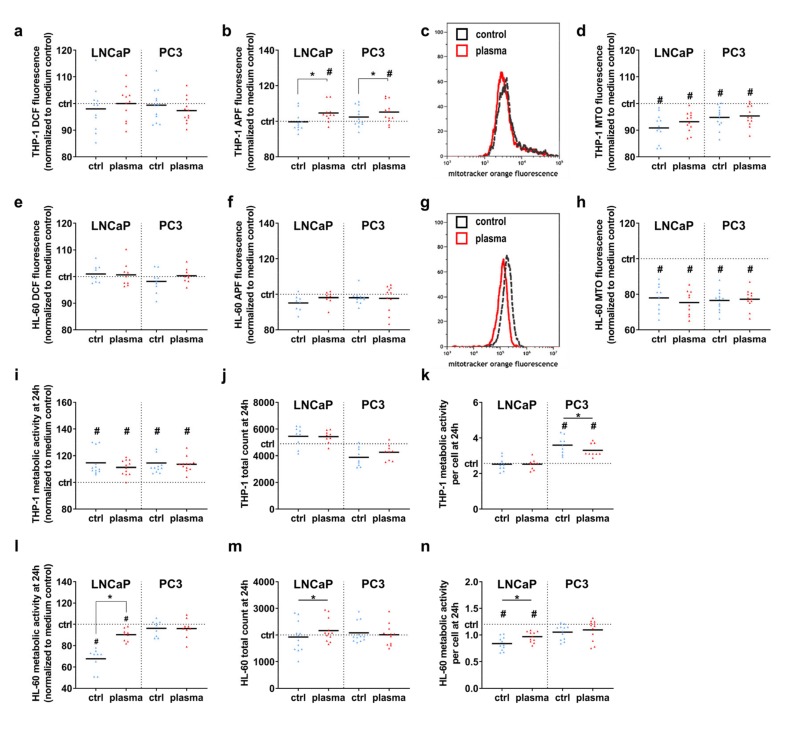
Oxidation and metabolic activity of myeloid cells following exposure to supernatants of kINPen plasma-treated prostate cancer cells. (**a**,**b**) normalized mean fluorescent intensity of THP-1 cells stained with (**a**) DCF and (**b**) APF, two dyes indicating intracellular redox changes, and exposed to control cell culture medium (dashed line), untreated supernatants of LNCaP or PC3 cells (ctrl), or supernatants of plasma-treated LNCaP or PC3 cells (plasma); (**c**,**d**) overlay histogram (**c**) and quantification (**d**) of THP-1 cells stained with mitotracker orange (MTO) and exposed to control cell culture medium (dashed line), untreated supernatants of LNCaP or PC3 cells (ctrl), or supernatants of plasma-treated LNCaP or PC3 cells (plasma); (**e**,**f**) normalized mean fluorescent intensity of HL-60 cells stained with (**e**) DCF and (**f**) APF, and exposed to control cell culture medium (dashed line), untreated supernatants of LNCaP or PC3 cells (ctrl), or supernatants of plasma-treated LNCaP or PC3 cells (plasma); (**g**,**h**) overlay histogram (**g**) and quantification (**h**) of HL-60 cells stained with mitotracker orange (MTO) and exposed to control cell culture medium (dashed line), untreated supernatants of LNCaP or PC3 cells (ctrl), or supernatants of plasma-treated LNCaP or PC3 cells (plasma); (**i**–**k**) metabolic activity (**i**) and absolute cell counts (**j**) as well as ratio of metabolic activity over cell counts (**k**) of THP-1 cells exposed to control cell culture medium (dashed line), untreated supernatants of LNCaP or PC3 cells (ctrl), or supernatants of plasma-treated LNCaP or PC3 cells (plasma) at 24 h; (**l**–**n**) metabolic activity (**l**) and absolute cell counts (**m**) as well as ratio of metabolic activity over cell counts (**n**) of HL-60 cells exposed to control cell culture medium (dashed line), untreated supernatants of LNCaP or PC3 cells (ctrl), or supernatants of plasma-treated LNCaP or PC3 cells (plasma) at 24 h. Data are presented as mean of three to five independent experiments with several technical replicates each. Statistical analysis was performed using ratio-paired t-test. ctrl = control. The level of significance is indicated as follows: *p* < 0.05 (*) or (#).

The myeloid cell lines THP-1 and HL-60 were incubated with regular cell culture medium, supernatants from untreated prostate cancer cells, or supernatants from plasma-treated prostate cancer cells. Many cellular processes are subject to redox control, and we investigated the intracellular oxidative milieu of the myeloid cells using the redox-sensitive dyes DCF and APF that accumulate in the cytosol of cells upon staining. DCF is a pan-ROS sensor [33], while a certain degree of specificity towards three types of ROS (hypochlorous acid, peroxynitrite, and hydroxyl radical) is attributed to APF [34]. In THP-1 cells, the plasma-treated supernatants did not change DCF (Figure 2a) but APF intensities, indicative of more intracellular ROS (Figure 2b). The difference was significant when compared against both the use of untreated cancer cell supernatants and medium control only. Mitochondria can be a source of intracellular ROS following response to a stimulus. To this end, the mitochondrial membrane potential was investigated (Figure 2c) and a significant decrease was observed in response to incubation with all types of prostate cancer cell supernatants when compared against the medium control (Figure 2d). In HL-60 cells, and similarly to THP-1, DCF did not show significant changes in fluorescence for any of the conditions investigated (Figure 2e). In contrast to THP-1 cells, this was also the case for APF in HL-60 cells (Figure 2f). For mitochondria, however, a decrease in the membrane potential was observed (Figure 2g), which was significant following incubation with all types of prostate cancer cell supernatants when compared against the medium control (Figure 2h). These data suggest that the tumor cell supernatants are sensed and recognized by the myeloid cells, as evident in the mitochondria response and partially also in the ROS production. Mitochondrial activity is closely linked to metabolic activity. To this end, the effect of the cancer cell supernatants on the metabolic activity and proliferation of myeloid cells was investigated 24 h after incubation. For THP-1 cells, a significant increase in metabolic activity was observed (Figure 2i), while cell numbers differed only to a minor extent from that of the cell culture medium control (Figure 2j). The metabolic activity per cell was significantly increased for supernatants from PC3 cells (Figure 2k). For HL-60 cells, a decrease in metabolic activity was found, which was significant for supernatants from LNCaP cells (Figure 2l). The absolute cell numbers indicative of changes in proliferation did not vary to a great extent (Figure 2m), which in sum led to a significantly decreased metabolic activity per cell for supernatants from LNCaP on HL-60 cells (Figure 2n). In summary, it can be concluded for the metabolic activity assays that the supernatants of the prostate cancer cells had an impact on the myeloid cell activity, especially in THP-1 cells. The plasma treatment, however, did not provoke effects much different from those observed with supernatants from untreated prostate cancer cells.

### 3.3. Plasma-Treated Prostate Cancer Cell Supernatants Promoted Immunomodulation in Myeloid Cells

**Figure 3:**
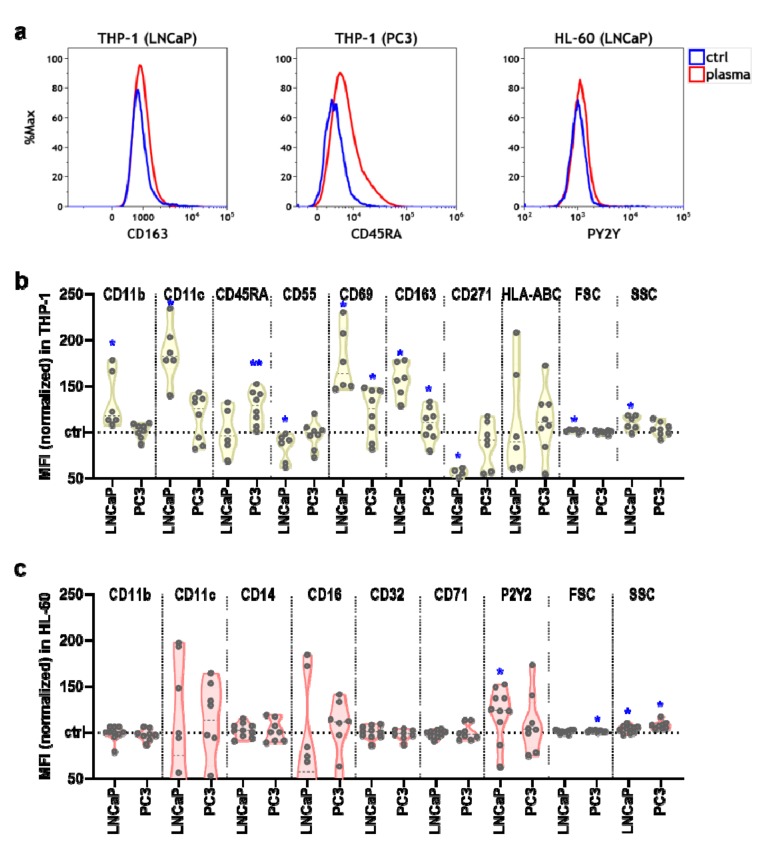
The cell surface marker profiles of myeloid cells following exposure to supernatants of kINPen plasma-treated prostate cancer cells. (**a**) representative overlay histograms of the flow cytometry analysis; (**b**) normalized mean fluorescent intensities at 96 h of CD11b, CD11c, CD45RA, CD55, CD69, CD163, CD271, HLA-ABC, forward scatter, and side scatter of THP-1 cells exposed to untreated supernatants of LNCaP or PC3 cells (ctrl, dashed line) or supernatants of plasma-treated LNCaP or PC3 cells; (**c**) normalized mean fluorescent intensities at 96 h of forward scatter, side scatter, CD11b, CD11c, CD14, CD16, CD32, CD71, and P2Y2 of HL-60 cells exposed to untreated supernatants of LNCaP or PC3 cells (ctrl, dashed line) or supernatants of plasma-treated LNCaP or PC3 cells. Data are presented as violin plots with median of three to five independent experiments with several technical replicates each. Statistical analysis was performed using ratio-paired t-test. The level of significance is indicated as follows: *p* < 0.05 (*) and *p* < 0.01 (**).

The activity and differentiation of immune cells can be judged based on their expression of surface molecules and the release of pro and anti-inflammatory mediators such as chemokines and cytokines. Tumor cells secrete immunomodulatory mediators into the liquid environment. It was hypothesized that plasma treatment changes the composition and concentration of these mediators, which would lead to a different myeloid cell phenotype. To this end, we investigated both the surface receptor expression profile and chemokine and cytokine release of myeloid cells cultured with supernatants of untreated or plasma-treated prostate cancer cells at 96 h. Representative overlay flow cytometry histograms illustrate the distribution of a selection of surface receptor staining intensities (Figure 3a). For THP-1 cells, there was a significant increase observed with supernatants of plasma-treated over untreated prostate cancer cells for the cell surface markers CD11b, CD11c, and CD45RA (Figure 3c), pointing to increased activation of the cells. Other significant changes observed were a decrease for CD55, an increase in CD69, an increase in CD163, a decrease in CD271, and an increase in both cell size and granularity, while the expression of HLA-ABC was unchanged. Most changes were observed for supernatants of LNCaP (8 out of 10 markers), while significant changes with PC3 cell supernatants were less prominent (2 out of 10 markers). For HL-60 cells, incubation with supernatants of plasma-treated prostate cancer cells increased cell size and granularity but not the expression of CD11b, CD11c, CD14, CD32, and CD 71 (Figure 3c). For P2Y2, a significant increase was observed for plasma-treated LNCaP cells and their supernatants. In general, the markers investigated are indicators of myeloid cell activation and differentiation, and the effect of the supernatants was more pronounced in THP-1 compared to HL-60 cells.

**Figure 4:**
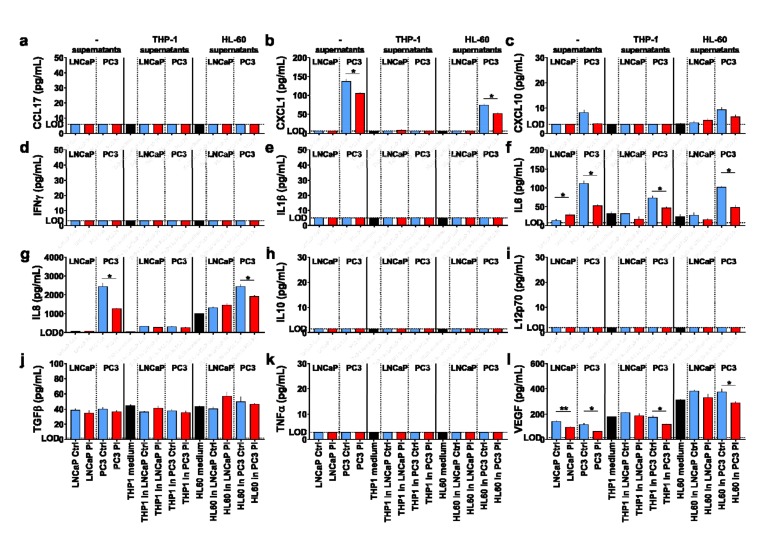
The cytokine profiles of myeloid cells following exposure to supernatants of kINPen plasma-treated prostate cancer cells. (**a**–**l**) quantification of the levels of CCL17 (**a**), CXCL1 (**b**), CXCL10 (**c**), IFNγ (**d**), IL1β (**e**), IL6 (**f**), IL8 (**g**), IL10 (**h**), IL12p70 (**i**), TGFβ (**j**), TNFα (**k**), and vascular endothelial growth factor (VEGF) (**l**) in untreated and kINPen plasma-treated prostate cancer cells (collected at 4 h) as well as THP-1 and HL-60 cell culture supernatants at 96 h with or without the addition of untreated or kINPen plasma-treated tumor cell culture supernatants. Data show the mean + S.E. of data from supernatants retrieved from at least three independent experiments. Statistical analysis was performed using ratio-paired *t*-test. The level of significance is indicated as follows: *p* < 0.05 (*) and *p* < 0.01 (**). ctrl = control; Pl = plasma; LOD = limit of detection.

To elucidate the immunomodulatory potential of the supernatants further, 12 different chemokines and cytokines were quantified in the prostate cancer cell as well as myeloid cell supernatants (Figure 4). This included CCL17 (Figure 4a), CXCL1 (Figure 4b), CXCL10 (Figure 4c), IFNγ (Figure 4d), IL1β (Figure 4e), IL6 (Figure 4f), IL8 (Figure 4g), IL10 (Figure 4h), IL12p70 (Figure 4i), TGFβ (Figure 4j), TNFα (Figure 4k), and VEGF (Figure 4l). For LNCaP, only IL6, IL1β, and VEGF were detectable, while for PC3 cells, CXCL1, CXCL10, and IL8 were also found. Plasma treatment significantly decreased the levels of all targets released from PC3 cells, while for LNCaP, a decrease in IL8 and VEGF, and an increase in IL6 was observed. VEGF is a tumor-promoting factor [35], and its decrease with plasma exposure points to a potentially beneficial effect. For THP-1 and HL-60 cells alone without tumor cell supernatants, only IL6, IL8, TGFβ, and VEGF were found. For the supernatants of plasma-treated vs. control prostate cancer cells alone (not added to myeloid cells), a significant decrease was found for IL6 and VEGF. The same was found for HL-60 cells, with other significant decreases observed for CXCL1 and IL8. Notably, there was no significant difference for supernatants of LNCaP cells incubated on myeloid cells. This is in line with the surface marker expression data for HL-60 but not THP-1 cells, with the latter showing a marked modulation that, however, was not reflected in the chemokine and cytokine spectrum.

### 3.4. Plasma-Treated Prostate Cancer Cells Promoted Myeloid Cell Differentiation and Tumor-Toxic Activity

**Figure 5:**
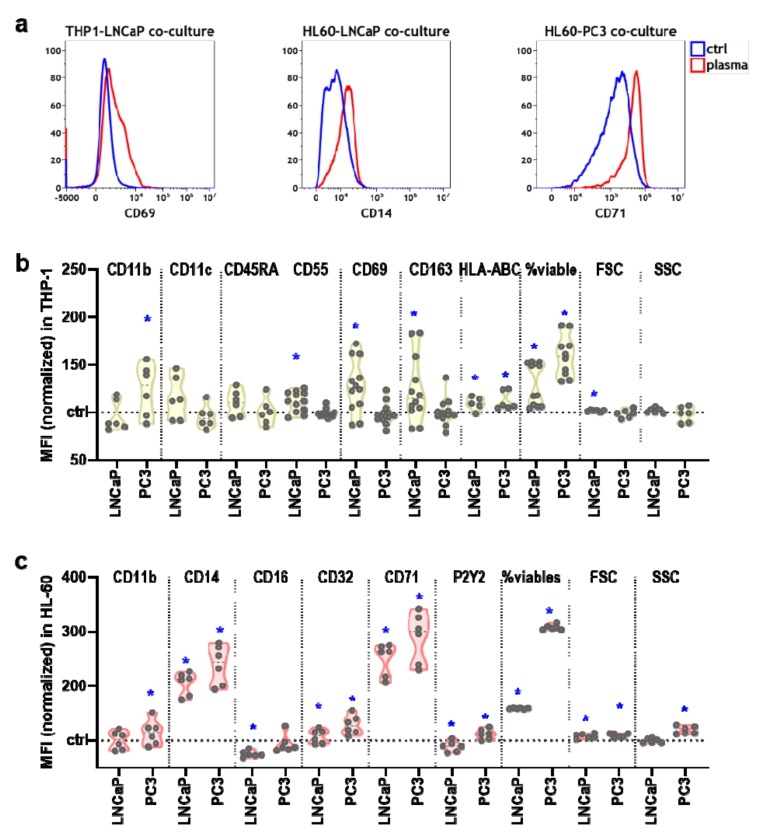
The cell surface marker profiles of myeloid cells co-cultured with kINPen plasma-treated prostate cancer cells. (**a**) representative overlay histograms of the flow cytometry analysis; (**b**) normalized mean fluorescent intensities at 96 h of CD11b, CD11c, CD45RA, CD55, CD69, CD163, HLA-ABC, viable cells, forward scatter, and side scatter of THP-1 cells cultured with either untreated (ctrl, dashed line) or plasma-treated LNCaP or PC3 cells; (**c**) normalized mean fluorescent intensities at 96 h of forward scatter, side scatter, CD11b, CD14, CD16, CD32, CD71, P2Y2, and viable HL-60 cells cultured with either untreated (ctrl, dashed line) or plasma-treated LNCaP or PC3 cells. Data are presented as violin plots and median of three to five independent experiments with several technical replicates each. Statistical analysis was performed using ratio-paired t-test. The level of significance is indicated as follows: *p* < 0.05 (*).

The supernatants of untreated and plasma-treated prostate cancer cells led to a modulation of intracellular ROS, metabolic activity, cell surface marker expression, and cytokine release in myeloid cells. The next question was to understand how the direct cell–cell interaction between plasma-treated tumor and myeloid cells induced immunomodulation in myeloid cells. Representative overlay flow cytometry histograms illustrate the distribution of selected signal intensities (Figure 5a). A range of cell surface receptors was investigated in THP-1 cells, including CD11b, CD11c, CD45RA, CD55, CD69, CD163, HLA-ABC, as well as the percentage of viable cells, and cell size and granularity (Figure 5b). Except for CD11c and granularity, a significant increase was observed for all markers for one or both of the prostate cancer cell co-cultures investigated. This was even more pronounced in HL-60 cells, where all of the studied parameters were significantly regulated in one or both of the prostate cancer cell co-cultures. This included cell viability, size, and granularity as well as expression of CD11b, CD14, CD16, CD32, CD71, and P2Y2 (Figure 5c). This suggested that the plasma-induced prostate cancer cell death was driving differentiation-like responses in human myeloid as compared to those observed in co-cultures with untreated prostate cancer cells.

**Figure 6:**
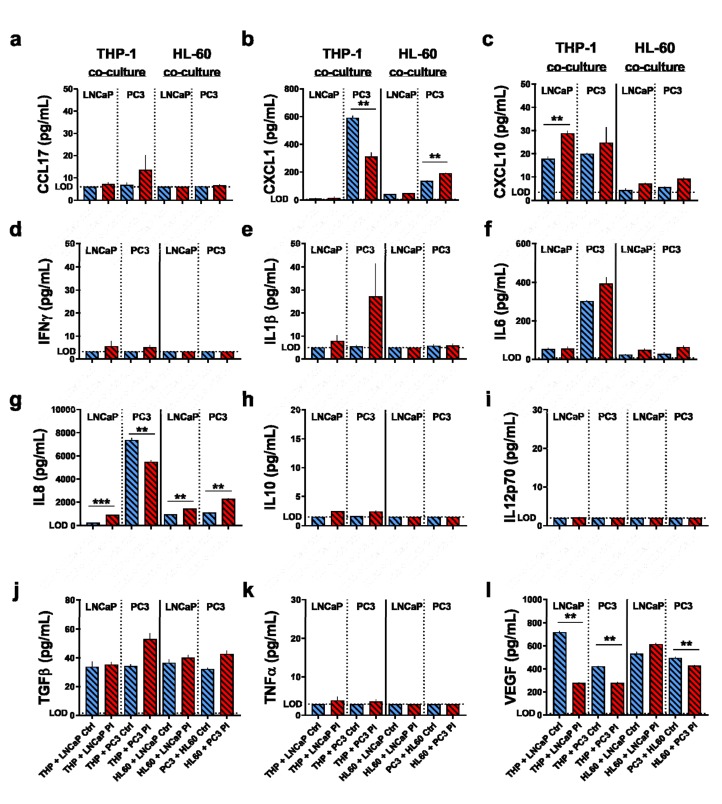
The cytokine profiles of myeloid cells co-cultured with kINPen plasma-treated prostate cancer cells. (**a**–**m**) quantification of the levels of CCL17 (**a**), CXCL1 (**b**), CXCL10 (**c**), IFNγ (**d**), IL1β (**e**), IL6 (**f**), IL8 (**g**), IL10 (**h**), IL12p70 (**i**), TGFβ (**j**), TNFα (**k**), and VEGF (**l**) retrieved after 96 h from supernatants of THP-1 and HL-60 cell culture supernatants co-cultured with untreated or kINPen plasma-treated prostate cancer cells. Data show the mean + S.E. of data from supernatants retrieved from at least three independent experiments. Statistical analysis was performed using ratio-paired *t*-test. The level of significance is indicated as follows: *p* < 0.01 (**) and *p* < 0.001 (***). ctrl = control; Pl = plasma; LOD = limit of detection.

To investigate the immunomodulatory potential of the secretory products of plasma-treated prostate cancer cells on myeloid cells, chemokine, and cytokine quantification of the same targets as above (Figure 4) was performed (Figure 6). For THP-1 prostate cancer co-cultures, a significant regulation of CXCL1, CXCL10, IL8, and VEGF was observed with plasma treatment. For HL-60, CXCL1, IL8, and VEGF were significantly regulated. With the exception of IL6 but not CXL10 being significantly regulated (Figure 4), the co-culture results were mostly congruent with those of the supernatant cultures except that the concentrations identified were several-fold higher.

**Figure 7:**
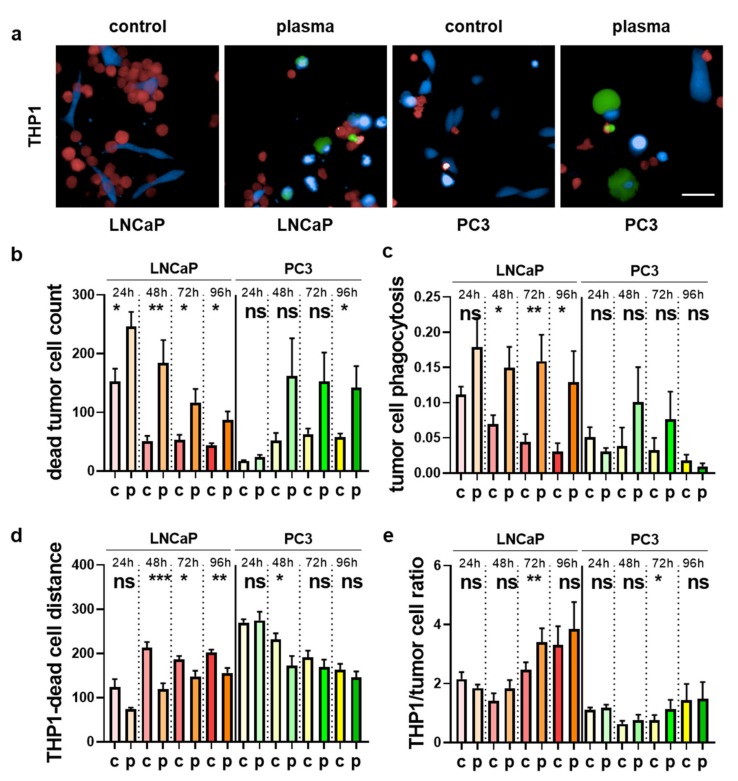
Quantitative image analysis of THP-1 cells co-cultured with plasma-treated prostate cancer cells. (**a**) prostate cancer cells were labeled with cell trace violet (blue) and were left untreated or exposed to the plasma, followed by addition of cell trace red (red) labeled THP-1 cells one hour later with sytox green (green) serving as dead cell marker; (**b**–**e**) algorithm-driven quantitative image analysis per image area of the total number of dead tumor cells per well (**b**), tumor cell phagocytosis (**c**), mean distance (in µm) between THP-1 cells and tumor cells (**d**), and the THP-1/tumor cell ratio (**e**). Data are presented as mean + S.E. of three independent experiments. Statistical analysis was performed using *t*-test. The level of significance is indicated as follows: *p* < 0.05 (*), *p* < 0.01 (**), and *p* < 0.001 (***). Scale bar is 50 µm. c = control; p = plasma; ns = not significant.

**Figure 8:**
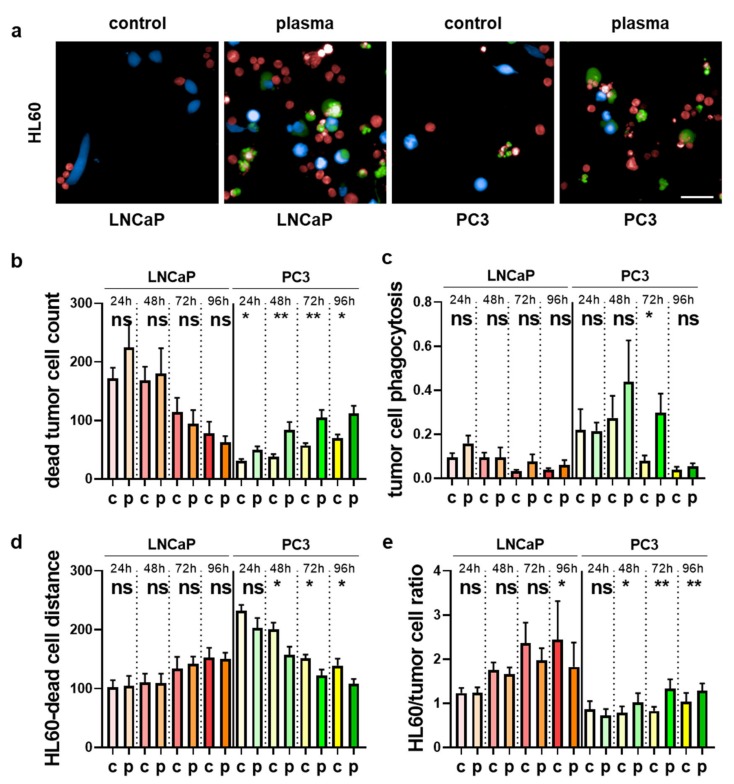
Quantitative image analysis of HL-60 cells co-cultured with plasma-treated prostate cancer cells. (**a**) prostate cancer cells were labeled with cell trace violet (blue) and were left untreated or exposed to the plasma, followed by addition of cell trace red (red) labeled HL-60 cells one hour later with sytox green (green) serving as dead cell marker; (**b**–**e**) algorithm-driven quantitative image analysis per image area of the total number of dead tumor cells per well (**b**), tumor cell phagocytosis (**c**), mean distance (in µm) between HL-60 cells and tumor cells (**d**), and the HL-60/tumor cell ratio (**e**). Data are presented as mean + S.E. of three independent experiments. Statistical analysis was performed using *t*-test. The level of significance is indicated as follows: *p* < 0.05 (*) and *p* < 0.01 (**). Scale bar is 50 µm. c = control; p = plasma; ns = not significant.

Plasma-treated prostate cancer cells co-cultured with myeloid cells spurred the expression of activation markers and modulated the cytokine release profiles. The next question was how these observed findings related to the tumor-toxic behavior of the myeloid cells. To this end, we utilized differential fluorescent staining and live-cell imaging to investigate several parameters using algorithm-driven quantitative image analysis. For THP-1 cells co-cultured with untreated or plasma-treated prostate cancer cells (Figure 7a), a significant increase in dead tumor cells was observed for LNCaP and in tendency also for PC3 cells (Figure 7b). Plasma-induced cell death is usually found within the first 24 h of incubation, but the number of dead cells in co-cultures was elevated throughout the time course up to 96 h, arguing for an auto-amplification of the tumor-toxic effects by THP-1 cells. This is supported by the enhanced phagocytosis index of THP-1 cells taking up prostate cancer cells (Figure 7c), suggesting clearance of dead prostate cancer cells. To investigate the effect of plasma treatment on the cell–cell interaction between the myeloid and prostate cancer cells, the mean distance between myeloid cells and cancer cells was calculated using algorithm-driven quantitative analysis. A decrease was observed (Figure 7d), indicating that the myeloid cells migrated closer to the tumor cells if the latter had been plasma-treated. We also calculated the THP-1 over the prostate cancer cell ratio to model growth and survival behavior in the co-cultures over the 96 h (Figure 7e). An increase for the THP-1 to tumor cell ratio was observed for the plasma treatment conditions that was significant for the 72 h time point. The same set of experiments was performed for HL-60 cells (Figure 8a). Here, a continuous elevation of dead tumor cells was less pronounced as compared to the results obtained for THP-1 cells, but significantly elevated cell death was observed for PC3 cells (Figure 8b). Tumor cell phagocytosis was increased in tendency in all conditions, being significant only for 72 h in co-cultures with PC3 cells (Figure 8c). The HL-60 to tumor cell distance was unchanged for LNCaP but significantly decreased for PC3 cells, pointing to enhanced migratory activity of HL-60 to PC3 cells. Lastly, the HL-60 to tumor cell ratio was decreased for HL-60-LNCaP co-cultures in the plasma conditions (Figure 8d), suggesting a growth advantage of the cancer cells over the myeloid cells in this setting. For plasma-treated PC3 cells, however, a significant increase of that ratio was observed (Figure 8e). From the results and the amplitude of changes observed, it can be concluded that THP-1 cells actively responded to plasma-treated LNCaP cells and mostly also to PC3 cells in terms of favorable repression of tumor cell growth and survival. By contrast, HL-60 cells seemed not to propagate the cytotoxic plasma effects when co-cultured with plasma-treated LNCaP cells, while responses to PC3 prostate cancer cells were more pronounced.

## 4. Discussion

The aim of this study was to investigate the immunomodulatory effects of two human plasma-treated prostate cancer cell lines towards two human myeloid cell lines in vitro. In comparison to untreated prostate cancer cells, the plasma treatment provoked significant changes in surface marker expression, cytokine release, and cytotoxic activity of the myeloid cells towards the tumor cells.

Supernatants of and co-cultures with cancer and myeloid cells modulated the surface marker expression and cytokine release upon plasma treatment. Nearly all markers investigated were significantly changed in at least one plasma-condition. Similar to CD11b [36,37], CD11c is associated with myeloid cell maturation in both cell types [38,39]. Interestingly, CD11c expression is regulated via AP-1 [40], a transcription factor we have recently described to respond to plasma-derived redox modulation in THP-1 cells [41]. Similarly, de novo CD69 expression is induced during the early activation of myeloid cells [42]. An increase in HLA-ABC expression, and cell size and granularity has been described for myeloid cell activation as well [39,43,44]. In terms of polarization, our marker panel was not designed for a detailed view on this aspect. We found CD163 to be increased in THP-1 cells, a marker commonly associated with M2 macrophages [45] but also found in inflammatory macrophages [46]. For HL-60 cells, a marked upregulation of CD14, CD32, and CD71 was determined only in co-culture experiments but not in the supernatant conditions related to plasma treatment. CD14, part of the LPS receptor, is associated with activation and maturation of HL-60 cells [47,48]. The same is true for CD32 [49,50], an Fcγ receptor. The transferrin receptor CD71, however, usually is internalized in activated HL-60 cells [51], leading to a decrease in surface receptor expression. Nevertheless, CD71 was also reported to be associated with macrophage development [52,53]. Although not all markers were regulated in each of the settings to a similar extent, a clear tendency towards activation was seen for both the supernatant and co-culture experiments. This suggests that plasma-induced cytotoxicity in prostate cancer cells leads to myeloid cell activation and differentiation, which often is also associated with changes in the secretion profile of cytokines [54].

Cytokine release reflects the inflammatory milieu generated by cells. In the supernatant experiments, a significant decrease was observed for CXCL1, IL6, IL8, and VEGF in the plasma conditions. Both IL6 and IL8 are markers of activation and differentiation in myeloid cells, at least when using mitogens as stimulating agents [55]. Along similar lines, an increase in CXCL1 is found in M1 macrophages differentiated with PMA [56]. Increased VEGF is observed in M2 polarized macrophages [57]. This suggests that activation and differentiation were lower with plasma treatment of tumor cells. However, our data also show that the levels of CXCL1, IL6, IL8, and VEGF for myeloid cells were similar to that of the tumor cells alone. Hence, it can be speculated that the contribution of the myeloid cells to the cytokine levels was not pronounced in the supernatant experiments, despite the significant changes in surface marker expression. One reason for that might be the low initial cell number used, and the ability of the cells to internalize extracellular chemokines and cytokines produced during the 96 h-period of incubation. The unchanged levels of TGFβ and the significantly decreased levels of VEGF need to be emphasized because both are potent tumor-promoting effectors in the tumor microenvironment [58,59]. This was also observed in our co-culture experiments, pointing to a potentially beneficial effect of the plasma treatment. In contrast to the supernatant experiment, a marked increase for CXCL10 was observed in the plasma conditions. CXCL10 is released upon tumor-toxic M1 macrophage polarization [60]. IL8 also was significantly increased in three of the four plasma-conditions investigated in the co-culture system, and the chemokine is associated with chemo-attraction of phagocytes and increased inflammation. Several other pro-inflammatory targets leading to potentially tumor-toxic effects, such as CCL17, IFNγ, IL1β, and IL6, were increased in tendency. It was previously reported that myeloid cell-derived IL1β abrogated proliferation of LNCaP cells [61]. Elevated IL6 levels may also be a consequence of a feedback loop between cancer and myeloid cells, as least for LNCaP and THP-1 cells [62]. Our data on only little induction of IL1β and IL6 in myeloid cells by prostate cancer cell-derived supernatants are in line with previous findings [63]. A noticeable increase in TNFα, as identified in a previous study being the principal tumor-toxic agent of macrophages [64], was not observed, however. In general, it is still a matter of debate of how macrophages exert cytotoxic effects against cancer cells. Besides the reported TNFα [63], activated M1 but not M2 macrophages were previously found to produce family 18 chitinases that pose potent tumor-toxic acitivity [65]. GM-CSF was also noted to enhance the tumor toxic activity of macrophages in vitro [66], while others reported direct antibody-dependent cytotoxicity and phagocytosis of tumor cells as mechanisms of action [67].

Nevertheless, we observed cytotoxic effects in prostate cancer cells co-cultured with myeloid cells that extended much further to the initial plasma treatment at 0 h. This was supported by our findings on increased tumor cell uptake and migration towards tumor cells in the plasma conditions. Enhanced tumor-toxicity in co-culture systems was reported before with activated vs. non-activated THP-1 cells [68]. In our study, we have several indices of macrophage activation through direct interaction with plasma-treated prostate cancer cells and their secretory products. At least in THP-1 cells, which showed greater tumor-toxic properties compared to HL-60 cells, this was underlined by an increase in intracellular ROS, a hallmark of monocyte activation [69] and macrophage differentiation [70]. Both myeloid cell lines showed a significant decrease in the mitochondrial membrane potential, which is associated with increased ROS production [71]. Whether the differences observed with DCF and APF point to differences in ROS entities and functional consequences in myeloid cells remains an open question. Notwithstanding, especially the HL-60-LNCaP co-culture showed the least cytotoxic activity on the cancer cells following plasma exposure. From the cell surface and cytokine data, it seems that this condition did not promote HL-60 differentiation and activation to such an extent as observed for PC3 cells, for instance. CD11b, CD14, CD32, CD71, and side scatter were markedly less increased in co-culture with LNCaP compared to PC3 cells, which supports this view. CD16, a marker of differentiation in HL-60 cells [72], even was significantly decreased in HL-60 cells co-cultured with plasma-treated LNCaP over untreated LNCaP cells. Moreover, this co-culture condition was the only one among the four myeloid cell–tumor cell co-cultures that showed a non-significant increase instead of a significant decrease in VEGF. VEGF can polarize myeloid cells towards a non-inflammatory phenotype [73], which might explain the lower killing activity of HL-60 cells in co-culture with the LNCaP cells.

Our study focused on myeloid cell activation and inflammatory markers in response to plasma-killed prostate cancer cells. Monocyte-to-macrophage differentiation and polarization, mainly to tumor-associated macrophages (TAM), is a clinical trait of prostate cancer associated with poor prognosis and tumor recurrence [74,75]. Mechanistically, tumors constantly attract myeloid cells from the bloodstream via several secreted factors, such as VEGF, which in turn drives pro-tumorigenic macrophage polarization [76,77]. Plasma treatment decreased VEGF levels in prostate cancer cell supernatants and co-cultures with myeloid cells. This suggests that plasma treatment might be able to modulate the tumor microenvironment in disfavor of tumor growth. This question should be targeted in future studies using more complex prostate tumor models and primary myeloid cells, both being a limitation of our study. Nevertheless, THP-1 cells are a good model for studying monocyte activation and differentiation, and compared genereally well with primary monocytes and especially M1 polarization in various previous studies [78,79,80,81,82,83]. Another question is how plasma treatment might be applied clinically in prostate cancer patients as already done in a fraction of head and neck cancer patients [84,85]. One option would be to expose to resection-margins after surgery to the gas plasma. This way, micro-metastasis that often lead to tumor recurrence might be decreased. This might also be achieved using plasma-treated liquids that were recently proposed to have anticancer properties as well [86,87]. Such liquid might also be injected directly into the tumor, using guided ultrasound microinjection. Tumor-toxic injections with plasma-treated liquids have been recently demonstrated in vivo using a model of pancreatic cancer [88]. Finally, another timely question is whether plasma treatment might be able to revert chemotherapeutic resistance, but apart from first studies on drug-transporter expression in cancer cells after plasma treatment [89,90], such studies are yet awaited to come.

## 5. Conclusions

Plasma-treated prostate cancer cells were superior in activating myeloid cells as compared to the tumor cell supernatants. This suggests that not only soluble factors but also direct cell–cell contact to be a factor promoting the cytotoxicity and differentiation of tumor-toxic myeloid cells. The promising nature of these findings in terms of gas plasma treatment of prostate cancer needs to be further evaluated in experimental disease models in the future.

## Figures and Tables

**Table 1:** Antibodies and clones used in this study.

Target	Clone	Vendor
CD11b	ICRF44	BD
CD11c	118/A5	eBioscience
CD14	TÜK4	Miltenyi Biotec
CD16	CLB-gran11.5	BD
CD32	6C4	eBioscience
CD45RA	HI100	BD
CD55	IA10	BD
CD69	FN50	BD
CD71	M-A712	BD
CD163	GHI/61	BD
CD271	C40-1457	BD
HLA-ABC	G46-2.6	BD
P2Y2	E-3	SantaCruz
